# Evaluation of the safety and efficiency of cytotoxic T cell therapy sensitized by tumor antigens original from T‐ALL‐iPSC in vivo

**DOI:** 10.1002/cai2.95

**Published:** 2023-10-19

**Authors:** Weiran Li, Meiling Zhou, Lu Wang, Liying Huang, Xuemei Chen, Xizhuo Sun, Tao Liu

**Affiliations:** ^1^ Department of Tumor Immunotherapy, Shenzhen Luohu People's Hospital The Third Affiliated Hospital of Shenzhen University Shenzhen Guangdong China; ^2^ Cell Quality Testing Laboratory of Shenzhen Luohu Hospital Group Shenzhen Guangdong China

**Keywords:** adoptive cell therapy, drug safety evaluation, IPSCs, T‐ALL

## Abstract

**Background:**

Since RNA sequencing has shown that induced pluripotent stem cells (iPSCs) share a common antigen profile with tumor cells, cancer vaccines that focus on iPSCs have made promising progress in recent years. Previously, we showed that iPSCs derived from leukemic cells of patients with primary T cell acute lymphoblastic leukemia (T‐ALL) have a gene expression profile similar to that of T‐ALL cell lines.

**Methods:**

Mice with T‐ALL were treated with dendritic and T (DC‐T) cells loaded with intact and complete antigens from T‐ALL‐derived iPSCs (T‐ALL‐iPSCs). We evaluated the safety and antitumor efficiency of autologous tumor‐derived iPSC antigens by flow cytometry, cytokine release assay, acute toxicity experiments, long‐term toxicity experiments, and other methods.

**Results:**

Our results indicate that complete tumor antigens from T‐ALL‐iPSCs could inhibit the growth of inoculated tumors in immunocompromised mice without causing acute and long‐term toxicity.

**Conclusion:**

T‐ALL‐iPSC‐based treatment is safe and can be used as a potential strategy for leukemia immunotherapy.

## Graphical abstract

We found that induced pluripotent stem cells (iPSCs) derived from leukemic cells of patients with primary T cell acute lymphoblastic leukemia (T‐ALL) have a gene expression profile similar to that of T‐ALL cell lines. Results indicate that complete tumor antigens from T‐ALL‐iPSCs could slow the progression of inoculated tumors in immunocompromised mice without causing acute and long‐term toxicity.

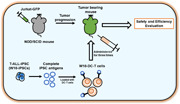

**Abbreviations**
ALT: alanine transaminase
AML‐iPSCs: iPSCs derived from acute myeloid leukemia patient samples
AST: aspartate aminotransferase
BUN: blood urea nitrogen
CAR‐T: chimeric antigen receptor T cell immunotherapy
CRE: creatinine
DC‐T: dendritic cells‐T cells
DiR: 1,1‐dioctadecyl‐3,3,3,3‐tetramethylindotricarbocyanine iodide
ESCs: embryonic cells
HE: hematoxylin and eosin staining
iPSCs: induced pluripotent stem cells
PBMCs: peripheral blood mononuclear cells
SD: standard deviation
T‐ALL: T cell acute lymphoblastic leukemia

## 1 INTRODUCTION

In the past few decades, immunotherapy has made significant progress. By 2020, the Center for Drug Evaluation (CDE), China had already accepted various applications for more than 99 cell therapy products. Two‐thirds of these were T cell‐based products. T cell adoptive cellular immunotherapies, including tumor‐infiltrating lymphocytes and genome‐edited T cell therapies, have shown great progress in basic and clinical research [1, 2, 3]. Owing to the TCR‐peptide‐major histocompatibility complex recognition mechanism, selecting a suitable therapy target is essential for prevention and treatment. Recently, safe and effective target antigens have been identified that could be used to achieve good clinical results for hematologic tumors [1, 4], multiple myeloma [5], liver cancer [6], gynecologic cancers [7], and others [8]. However, these therapies have limitations, and autogenous single‐target genome‐edited T cell therapies have a high cost, long treatment duration, and high risk, especially in clinical trials [9, 10].

Acute lymphoblastic leukemia (ALL) is a common hematological malignancy. Although adoptive T cell therapy has gained importance for the treatment of T cell acute lymphoblastic leukemia (T‐ALL), recurrence and drug resistance remain major clinical concerns [11]. Cancer heterogeneity and technical limitations have led to the ineffectiveness of single‐target genome‐edited T cell therapies. Studies have reported similarities in transcriptome features and antigens between various tumor cells and embryonic cells (ESCs) [12, 13]. Immunization with ESCs can lead to tumor rejection in mice [14]. Induced pluripotent stem cells (iPSCs) are alternative to ESCs and exhibit similar gene expression and surface marker profiles as ESCs [15, 16, 17, 18, 19, 20]. Moreover, iPSC‐based vaccines exhibit antitumor efficacy against breast cancer, mesothelioma, and melanoma [21].

iPSCs derived from tumor cells (such as gastrointestinal cancer cells, melanoma cells, and pancreatic ductal adenocarcinoma cells) have been widely used in cancer models and signaling pathways research [22, 23]. Studies have shown that iPSCs derived from acute myeloid leukemia (AML) patient samples (AML‐iPSCs) reset leukemic DNA methylation and gene expression patterns, but retain leukemic mutations [24, 25]. Another study showed that upon hematopoietic differentiation, AML‐iPSCs reproduce phenotypic and functional heterogeneity with all hallmarks of a leukemia stem cell hierarchy [26].

These studies suggest that using complete antigens of iPSCs derived from patient tumor cells could be more promising for targeting cancer. In our previous studies, we prepared several iPSC lines from the patient's peripheral blood mononuclear cells (PBMCs) and found that W10‐iPSCs [27] exhibit a gene expression profile similar to that of T‐ALL tumor cells [28]. Our in vitro experiments showed that dendritic and T (DC‐T) cells loaded with W10‐iPSC complete antigens (W10‐DC‐T) exhibit better killing effects on T‐ALL tumor cell lines compared to those loaded with iPSC antigens from healthy donors. To investigate the safety and effectiveness of this treatment, we established a T‐ALL immunodeficient mouse model and treated these animals with DC‐T cells loaded with T‐ALL iPSCs complete antigens. Results showed that this treatment successfully controlled the disease progression and did not cause acute or long‐term toxicity. Thus, treatment with DC‐T cells loaded with T‐ALL iPSCs complete antigens could be an efficient therapy to cure acute lymphocytic leukemia.

## 2 MATERIALS AND METHODS

### 2.1 iPSC generation and culture

In a previous study [27], we isolated PBMCs from patients with T‐ALL and healthy donors. iPSCs were derived from these PBMCs using the CytoTune®‐iPS 2.0 Sendai Reprogramming Kit (A16517; Thermo Fisher Scientific) and maintained in mTeSR Plus Basal Medium (05825; STEMCELL Technologies). L2‐iPSCs were generated from PBMCs of healthy donors and W10‐iPSCs were generated from PBMCs of patients with T‐ALL.

The human T‐ALL tumor cell lines Jurkat and Jurkat‐GFP were maintained in RPMI‐1640 (C11875500BT; Thermo Fisher Scientific) supplemented with 10% FBS (10099141C; Thermo Fisher Scientific).

All the cells were expanded and maintained at 37°C, 5% CO_2_.

Cell lysates were used for antigen loading. Briefly, cells (1 × 10^7^) were subjected to repeated freeze‐thaw cycles thrice in phosphate‐buffered saline using liquid nitrogen. After centrifugation, the protein concentration in the supernatant was determined using the Bradford Protein Assay kit (T9310A; Takara, Japan).

Dendritic cells (DCs) and T cells were isolated from PBMCs of healthy donors with low HLA‐A2 expression [28] and cultured using the DC‐CTL Cell Culture Kit (MCF‐001+MCF‐002; MoreCell). On Day 3 of the culture, immature DCs with treated with 30 µg/mL antigens and 1000 U/mL tumor necrosis factor‐α (TNF‐α) (C008; Nanoprotein). On Day 7, DCs were cocultured with T cells at a ratio of 1:5. The antigen‐loaded DC‐CTLs were then expanded in Alys‐505 complete culture media containing 30 IU/mL recombinant IL‐2 (C013; Nanoprotein).

### 2.2 Alkaline phosphatase (AP) and immunofluorescence staining

The VECTOR Blue AP Substrate kit (SK‐5300; Vector Laboratories) was used for AP staining according to the manufacturer's instructions. Following antibodies were used for immunofluorescence staining: rabbit anti‐Sox2 (1:400, A1193; ABclonal), Alexa Fluor 488‐labeled goat anti‐rabbit IgG (1:500, A0423; Beyotime), mouse anti‐SSEA4 (1:500, ab16287; Abcam), and Alexa Fluor 647‐labeled goat anti‐mouse IgG (1:500, A0473; Beyotime). 4′,6‐diamidino‐2‐phenylindole dihydrochloride (Sigma‐Aldrich) was used to stain the nucleus.

### 2.3 In vivo differentiation of iPSCs and histological analysis of teratomas

To confirm the pluripotency of T‐ALL iPSCs, 3–5 × 10^6^ cells were subcutaneously injected into nonobese diabetic/severe combined immunodeficiency (NOD/SCID) mice (age 6–10 weeks). Teratomas were harvested approximately 40 days after the injection and fixed in 4% paraformaldehyde. Teratoma sections were assessed using hematoxylin and eosin (HE) staining. Microscopic images were captured at ×40 magnification.

### 2.4 In vivo studies using the T‐ALL mouse model

To develop a mouse model of acute lymphatic leukemia, we intravenously injected Jurkat‐GFP cells into 6–10‐week‐old NOD/SCID mice. Briefly, on Day 0, 1 × 10^6^ Jurkat‐GFP cells were intravenously injected into each mouse. From Day 2, 2.5 × 10^6^ DC‐CTLs loaded with different types of antigens were intravenously administered to each mouse three times every 7 days. Body weight was measured every 3 days.

The in vivo distribution of antigen‐loaded DC‐T cells was analyzed by staining these cells with 1,1‐dioctadecyl‐3,3,3,3‐tetramethylindotricarbocyanine iodide (DiR; MB12482; Meilunbio). Briefly, DC‐T cells were labeled with 5 μM DiR for 20 min at 37°C and 5% CO_2_ before injection. Twenty‐four hours after intravenous administration, the mice were killed, and fluorescence images were captured using the Aniview in vivo Molecular Imaging system (Ex: 740 nm/Em: 820 nm).

### 2.5 Flow cytometric analysis

The peripheral blood cells of NOD/SCID mice were stained with PE‐anti‐human CD3 (PE‐hCD3, 300308; BioLegend), PE‐hCD45 (368510; BioLegend), PE‐hCD57 (359612; BioLegend), PE‐hTIM3 (345006; BioLegend), and APC‐anti‐mouse CD45 (103111; BioLegend), and then treated with ACK Lysis Buffer (420301; BioLegend). Flow cytometric assays were performed using the DxFLEX Flow Cytometer (Beckman).

### 2.6 Cytokine release assay

Cytokine levels in the plasma of NOD/SCID mice were examined using the LEGEND Plex Human Inflammation Panel (BioLegend) according to the manufacturer's instructions.

### 2.7 Evaluation of liver and kidney damage

Mouse peripheral blood was collected by submandibular vessel bleeding or cardiac puncture at the time of euthanasia. Heparin sodium was added to prevent blood clots. Blood samples were centrifuged at 800*g* for 10 min at 4°C, and plasma was collected and stored at −80°C. Plasma levels of creatinine (CRE), albumin, and blood urea nitrogen (BUN) were measured using testing kits (C011‐2‐1, A028‐2‐1, and C013‐2‐1, respectively; NjjcBio). Liver samples were homogenized by ultrasonication, and the levels of alanine transaminase (ALT) and aspartate aminotransferase (AST) were measured using the alanine aminotransferase (C009‐2‐1; NjjcBio) and AST (C010‐2‐1; NjjcBio) assay kits, respectively.

### 2.8 Statistical analysis

All data were analyzed using the one‐way analysis of variance and presented as the mean ± standard deviation. Differences were considered statistically significant at *p* < 0.05. All statistical analyses were performed using the GraphPad software.

## 3 RESULTS

### 3.1 CTLs loaded with complete antigens of T‐ALL‐iPSCs caused no acute toxicity in vivo

**Figure 1:**
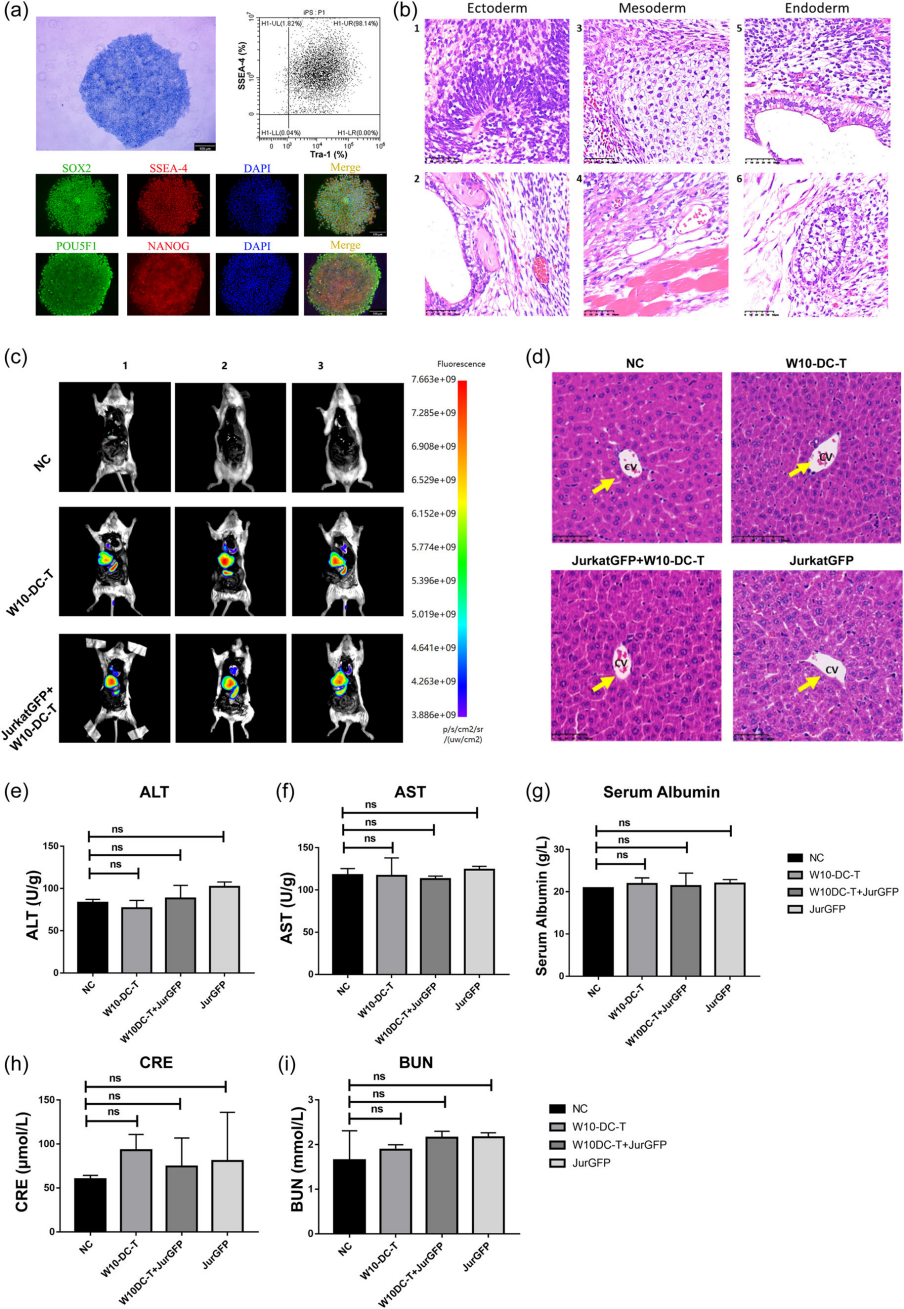
W10‐iPSC antigen‐loaded dendritic and T (W10‐DC‐T) cells did not induce acute toxicity in vivo. (a) Characterization of the pluripotent W10‐iPSCs using alkaline phosphatase staining (left), flow cytometry (right), and immunofluorescence staining (bottom). (b) Teratoma formation assay using W10‐iPSCs. Typical cells of the three germ layers were observed following hematoxylin and eosin (HE) staining. Scale bar, 50 μm. (c) The in vivo distribution of W10‐DC‐T cells after 24 h of intravenous administration. (d) HE staining showed that W10‐DC‐T cells did not cause liver damage. Scale bar, 50 μm. (e–g) The levels of alanine transaminase (ALT), aspartate aminotransferase (AST), and plasma albumin in different groups. (h and i) Creatinine (CRE) and blood urea nitrogen (BUN) levels in different groups. In (e–i), *n* = 3) for each group. DAPI, 4′,6‐diamidino‐2‐phenylindole. ns, no significance was compared with the negative control (NC) group.

Previously, we generated several iPSC lines from PBMCs of patients with T‐ALL [27]. Herein, we chose W10‐iPSCs, since RNA sequencing showed that the gene expression profile of W10‐iPSCs is similar to that of T‐ALL cell lines [28]. AP staining, flow cytometric analysis, and immunofluorescence staining were performed to characterize undifferentiated W10‐iPSCs (Figure 1a). W10‐iPSCs were subcutaneously injected into the NOD/SCID mice. HE staining of the W10‐iPSC‐derived teratoma confirmed the multipotent differentiation ability of these iPSCs (Figure 1b). Altogether, the results of our in vivo and in vitro studies showed that W10‐iPSCs are maintained in an undifferentiated state.

Complete antigens from W10‐iPSCs were loaded onto DC and T cells from healthy donors (W10‐DC‐T), which were then administered to T‐ALL‐bearing mice. Twenty‐four hours after intravenous administration, W10‐DC‐T cells tended to congregate in the liver of tumor‐bearing mice rather than in the spleen. In healthy mice, W10‐DC‐T cells were predominantly found in the spleen (Figure 1c). HE staining showed that W10‐DC‐T cells caused no structural damage to the liver (Figure 1d). Furthermore, the levels of ALT, AST, and plasma albumin were not significantly different among groups (Figure 1e–g). Plasma levels of CRE and BUN also showed no significant differences among groups (Figure 1h,i). Altogether, these results demonstrate that intravenous administration of W10‐DC‐T cells does not induce acute toxicity in healthy and tumor‐bearing mice.

### 3.2 W10‐DC‐T cell administration has no medium‐ and long‐term toxicity

**Figure 2:**
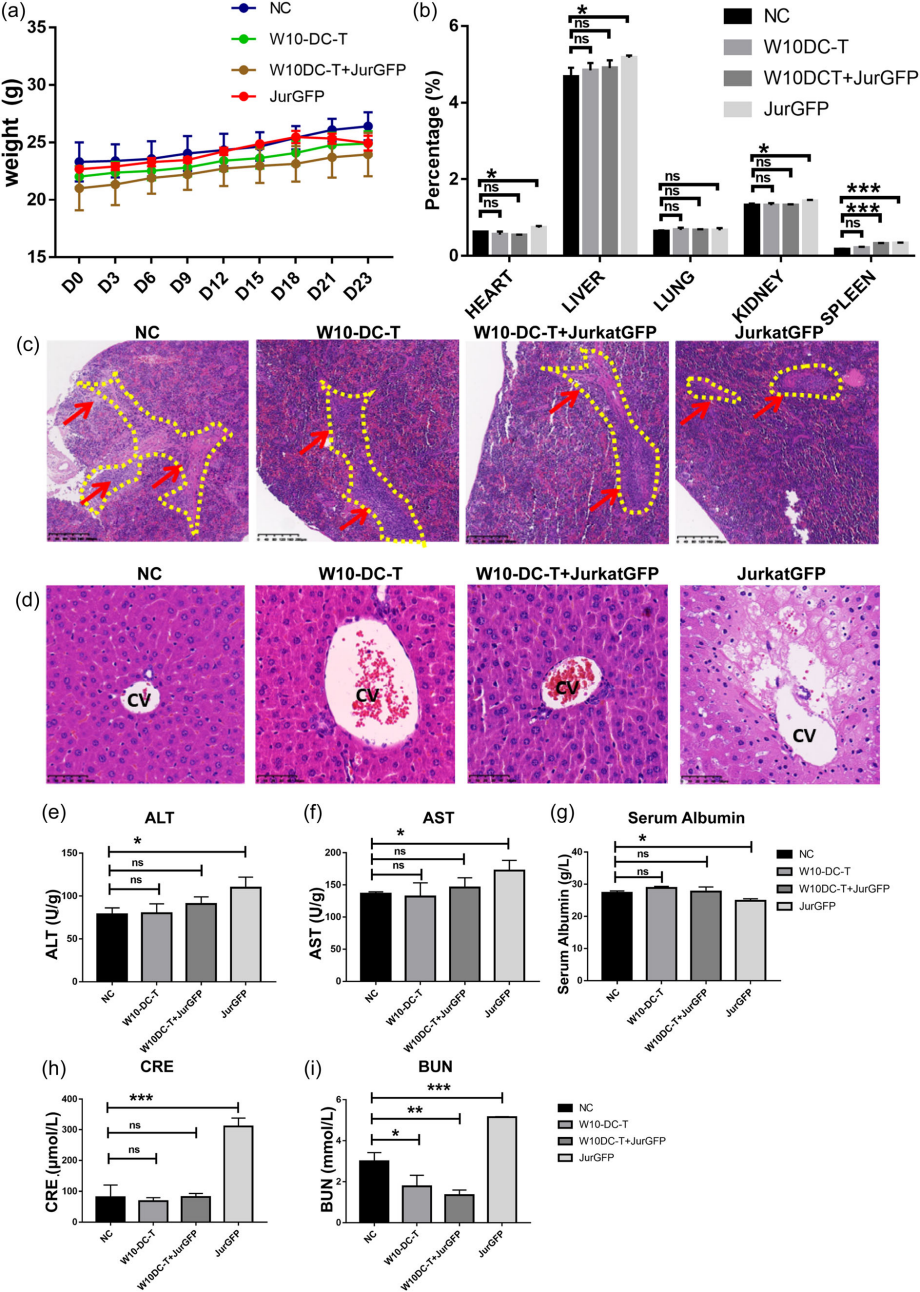
Thrice administration of W10‐iPSC antigen‐loaded dendritic and T (W10‐DC‐T did not induce toxicity. (a) Changes in the body weight of different group mice. Mice administered with W10‐DC‐T cells exhibited no change in body weight. *n* = 3. (b) Organ weight ratios in different groups. Administration of W10‐DC‐T cells increased the spleen weight in tumor‐bearing mice while ameliorating the weight gain of heart, liver, and kidneys caused by tumor burden. *n* = 3. (c and d) W10‐DC‐T cells relieved the compression of the white pulp and alleviated liver damage in tumor‐bearing mice. Scale bar, 50 μm. (e–g) The levels of alanine transaminase (ALT), aspartate aminotransferase (AST), and plasma albumin in different groups. (h and i) Creatinine (CRE) and blood urea nitrogen (BUN) levels in different groups. In (e–i), *n* = 3 for each group. All the groups were compared with negative control (NC), respectively. ns, no significance. *p < 0.05; **p < 0.01; ***p < 0.001.

We measured the body weight of mice in each group every day for 23 days after intravenous administration of W10‐DC‐T cells. No weight loss was observed in all groups except for the tumor‐bearing group without any T cell treatment (Jurkat‐GFP group; Figure 2a). HE staining showed that increased T‐ALL burden led to the compression of the white pulp of the spleen and necrosis of the hepatic lobules (Figure 2c,d). Increased levels of ALT, AST, and plasma albumin indicated liver damage due to tumor burden, while W10‐DC‐T cell treatment ameliorated this damage (Figure 2e–g). This protective effect of W10‐DC‐T cells was also observed in the kidney, as the levels of CRE and BUN in the Jurkat‐GFP group were higher (Figure 2h,i). In addition, the greater organ weight ratios of the heart, liver, and kidney may imply tumor burden damage, whereas W10‐DC‐T treatment brings them back to normal levels. Spleen weight was found to be increased in the W10‐DC‐T cell‐treated and Jurkat‐GFP groups, which might be due to the aggregation of T cells with tumor cells (Figure 2b). These results demonstrate that treatment with W10‐DC‐T cells reverses the damage caused by the tumor while inducing no medium‐ or long‐term toxicity.

### 3.3 W10‐DC‐T cell administration slowed the tumor progression

**Figure 3:**
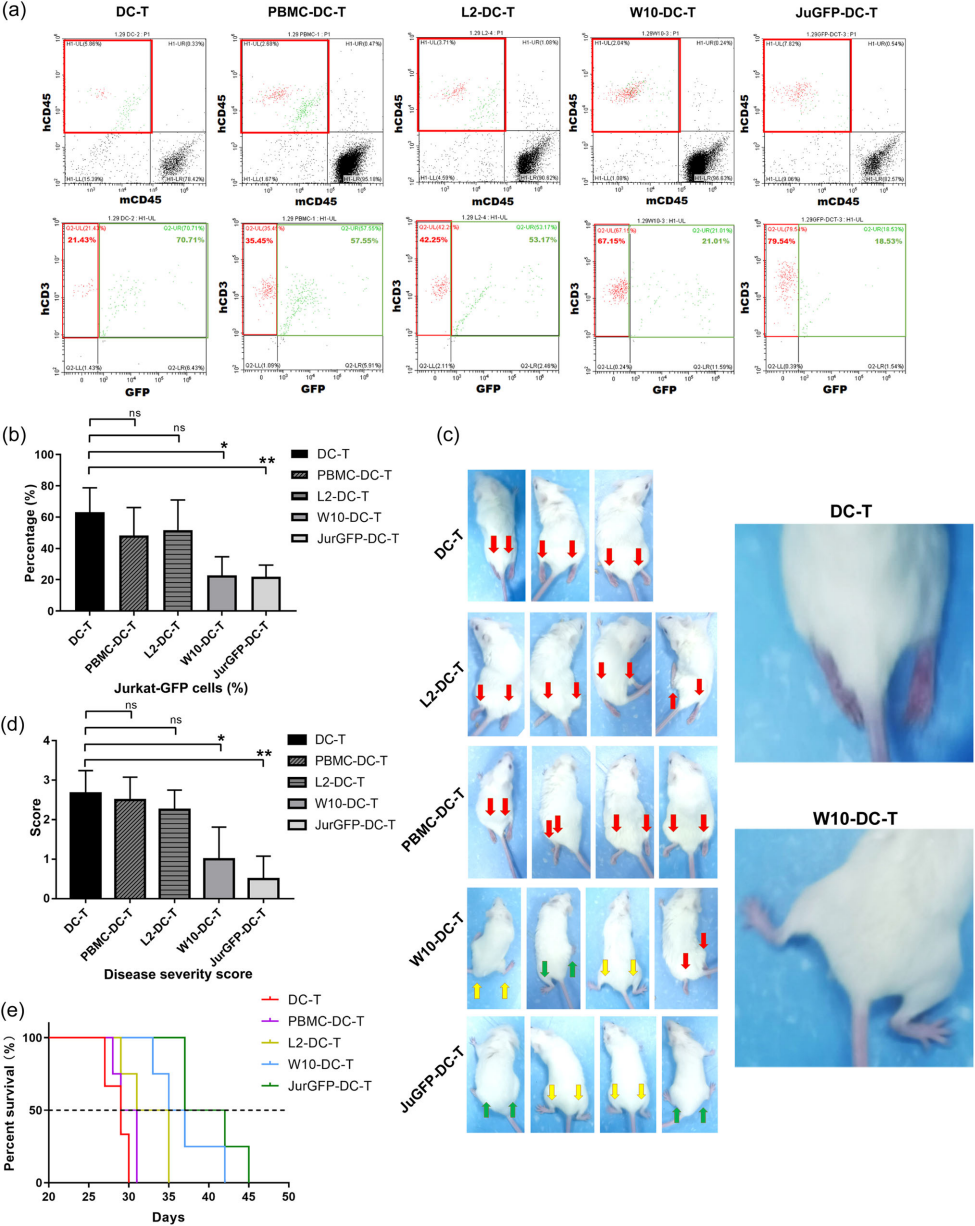
W10‐iPSC antigen‐loaded dendritic and T (W10‐DC‐T) cells exhibit antitumor efficacy in tumor‐bearing mice. (a) Flow cytometric analysis showed that W10‐DC‐T cell administration significantly decreased the proportion of Jurkat‐GFP cells. (b) Peripheral blood of nonobese diabetic/severe combined immunodeficiency (NOD/SCID) mice. (c and d) W10‐DC‐T cell administration significantly improved the function of the paretic hind limb. (e) W10‐DC‐T cell administration prolonged the survival of tumor‐bearing mice. ns, no significance; PBMC, peripheral blood mononuclear cell. *n* = 3. *p < 0.05 and **p < 0.01 were compared with negative control (NC).

**Figure 4:**
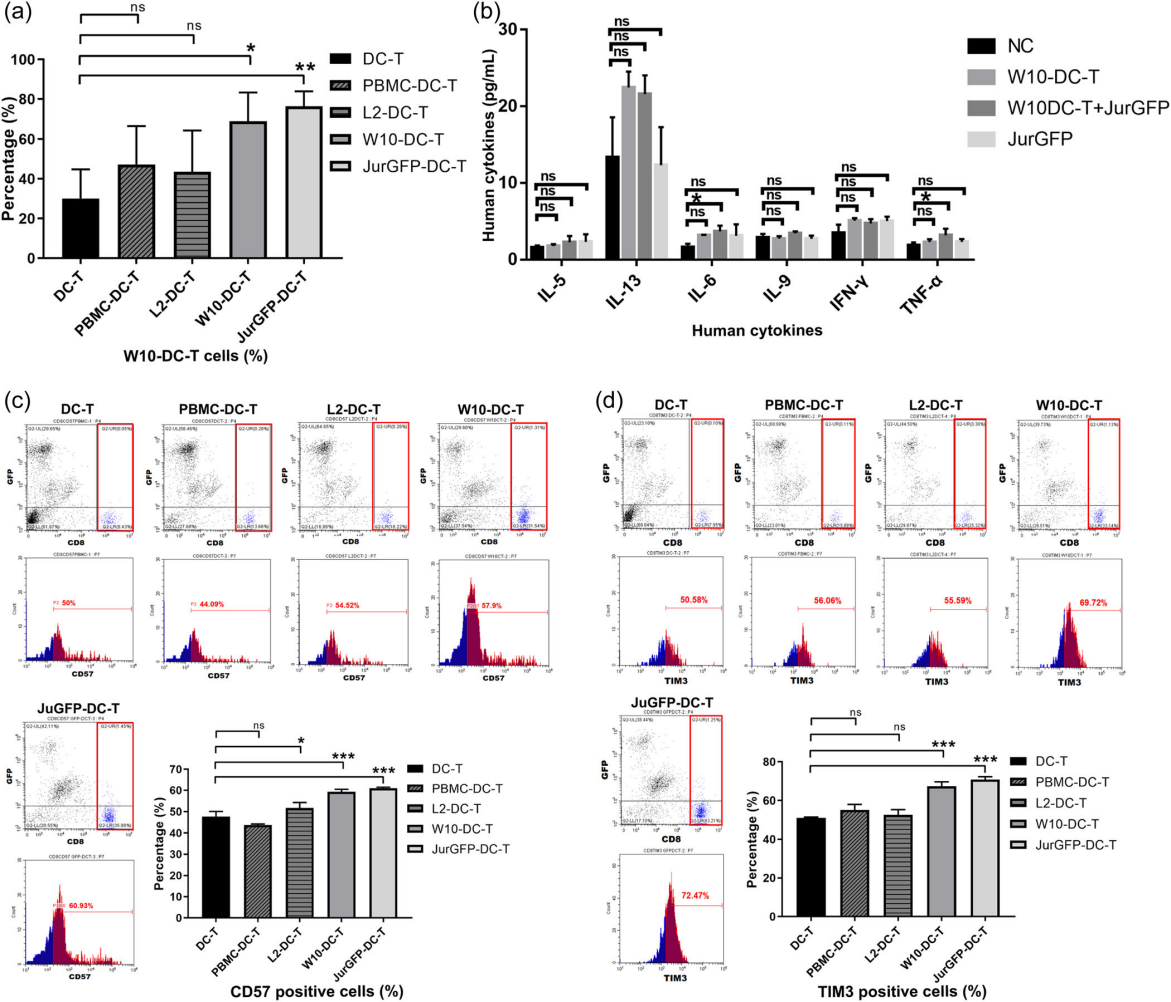
W10‐iPSC antigen‐loaded dendritic and T (W10‐DC‐T) cells can expand in vivo and cause cytotoxicity, leading to T cell aging. (a) The percentage of W10‐DC‐T cells in the peripheral blood of nonobese diabetic/severe combined immunodeficiency (NOD/SCID) mice. (b) The levels of human cytokines. W10‐DC‐T cells released proinflammatory cytokines (such as interleukin‐6 [IL‐6] and tumor necrosis factor‐α [TNF‐α]) in tumor‐bearing mice. (c and d) The surface expression of CD57 and TIM3 on T cells was increased in the W10‐DC‐T group. ns, no significance; PBMC, peripheral blood mononuclear cell. *n* = 3. *p < 0.05; **p < 0.01; ***p < 0.001.

To confirm the specific antitumor effects of W10‐DC‐T cells, we loaded DC‐T cells with several types of complete antigens (complete antigens of L2‐iPSCs and PBMCs were obtained from a healthy donor) and intravenously administered these cells to tumor‐bearing mice. After administering DC‐T cells twice, mouse peripheral blood was analyzed using flow cytometry (Figure 3a). The percentage of tumor cells in the W10‐DC‐T group was significantly lower than that in the L2‐iPS and negative control groups (Figure 3b). In addition, the percentage of human T cells was significantly higher in the W10‐DC‐T group (Figure 4a), implying the activation and expansion of T cells in vivo. On Day 23, untreated mice showed signs of disease. We rated the disease severity on a scale of zero to three. Mice with a score of zero had healthy and powerful hind limbs, those with a score of one had weaker hind limbs, and mice with a score of two were unable to stand or support their body. The mice with a score of three had dark and atrophied hind limbs, accompanied by urinary incontinence and paralysis (Figure 3c). Results showed that treatment with W10‐DC‐T cells slowed the progression of paralysis (Figure 3d) and prolonged the survival of tumor‐bearing mice (Figure 3e). These results demonstrated that W10‐DC‐T cell treatment successfully eliminated Jurkat‐GFP tumor cells and improved the condition of animals.

### 3.4 W10‐DC‐T cells release inflammatory factors in vivo and are prone to aging

After administering DC‐T cells thrice, we measured the levels of human cytokines and aging biomarkers of T cells. The levels of IL‐6 and TNF‐α were significantly higher in the W10‐DC‐T cell treatment group (W10‐DC‐T+Jurkat‐GFP) compared to the DC‐T control (W10‐DC‐T) and negative control (NC) groups (Figure 4b). As a potent proinflammatory cytokine, IL‐6 and IFN‐γ played a major role in cytokine release syndrome. Also, TNF‐α is a proinflammatory cytokine released by activated T cells. These results confirm the antitumor effects of W10‐DC‐T cells. However, we found that better antitumor effects were accompanied by T cell aging. Flow cytometric analysis showed that the percentage of CD57‐ and TIM3‐positive cells was significantly higher in the W10‐DC‐T group than that in the DC‐T group (Figure 4c,d). Higher concentrations of inflammatory cytokines always lead to deterioration of T cell function (T cell exhaustion) in chronic infections and cancer. Thus, this aging may associated with immune exhaustion.

## 4 DISCUSSION

DC‐based immunotherapy using complete tumor antigens [29] can elicit an immune response in all patients regardless of their HLA type [30, 31, 32, 33, 34]. Because of random mutations, full tumor antigens from individuals might be more potent; however, it is difficult to isolate primary tumor cells in the clinic. Our RNA‐sequencing results showed that cancer cells and ESCs/iPSCs share several tumor‐associated antigens. iPSC‐based vaccines can induce antitumor immunity [7, 35, 36, 37]. Autologous iPSCs can provide more accurate and representative tumor‐related antigens [38], thus opening up new possibilities [14, 39, 40].

Herein, we focused on hematological tumors to evaluate the antitumor efficiency of autologous tumor‐derived iPSC antigens. Since differentiation of iPSCs results in the loss of immunogenicity [41, 42], we used undifferentiated W10‐iPSCs. RNA sequencing showed that the gene expression profile of W10‐iPSCs is similar to that of T‐ALL cell lines. This provides the theoretical basis for this research. We found that DC‐T cells loaded with W10‐iPSCs complete antigens could reduce the hematologic Jurkat‐GFP tumor cell burden in NOD/SCID mice without inducing acute and prolonged toxicity. Moreover, since W10‐iPSCs can be stably cultured, it eliminates the problems related to ethical considerations and cell sources in the clinic.

Our study has some limitations. First, hematologic malignancies exhibit considerable histological and functional heterogeneity [43, 44]. Compared to the complete antigens of primary tumor cells, the complete antigens of single‐cell‐derived W10‐iPSCs have a narrower profile. Although W10‐iPSC antigens were efficient in animal models of T‐ALL, it remains unknown whether this treatment would inhibit tumorigenesis in patients with T‐ALL. Second, the potential long‐term toxicity of this treatment remains unknown. One published study on B7‐H4 chimeric antigen receptor T cell immunotherapy cell therapy reported delayed onset toxicity in NSG (NOD‐SCID gamma mouse) mice bearing OVCAR3 human ovarian cancer cell line xenografts [45]. No medium‐ or long‐term toxicity was observed in our study, although the possibility of delayed toxicity still exists. Fortunately, control switches (such as the caspase‐9 control switch) and TNF‐α blocking antibodies can stop the expansion and toxicity associated with excessive cytokine production by CAR T cells [46]. This suggests that the toxicity associated with T cell therapy could be managed using appropriate strategies. Furthermore, we also observed T cell aging and exhaustion in our study. In patients with chronic infection and cancer, persistent antigen stimulation results in T cell aging and exhaustion [47, 48]. Therefore, strategies should be developed to reactivate the patient's T cells effectively and improve the immune therapy responses. Finally, although the lifespan of tumor‐bearing mice was increased following the administration of W10‐DC‐T cells, the animals died once the treatment was stopped. Thus, the optimum administration dosage needs to be precisely determined.

## 5 CONCLUSIONS

In conclusion, we developed a novel cellular immunotherapy by loading complete iPSC antigens derived from primary tumor cells of patients with T‐ALL. This therapy successfully reduced the tumor burden, slowed the disease progression, and did not induce acute, medium, or long‐term toxicity in T‐ALL‐bearing NOD/SCID mice. Further studies should focus on the administration dosage and delayed‐onset toxicity.

## AUTHOR CONTRIBUTIONS

**Weiran Li**: Data curation (lead); writing—original draft (lead). **Meiling Zhou**: Data curation (equal); writing—original draft (equal). **Lu Wang**: Methodology (supporting); validation (supporting). **Liying Huang**: Methodology (supporting); validation (supporting). **Xuemei Chen**: Methodology (supporting); validation (supporting). **Xizhuo Sun**: Project administration (supporting); resources (supporting). **Tao Liu**: Project administration (lead); resources (lead).

## CONFLICT OF INTEREST STATEMENT

The authors declare no conflict of interest.

## ETHICS STATEMENT

The study was approved by the Ethics Committee of Shenzhen Luohu People's Hospital (LLBGS[2021]042) and conducted in accordance with applicable local regulations and the principles of the Declaration of Helsinki.

## INFORMED CONSENT

Written informed consent was obtained from the participants for use of blood samples.

## Data Availability

The data that support the findings of this study are available from the corresponding author upon reasonable request.
